# Advances in Imaging for Metastatic Epidural Spinal Cord Compression: A Comprehensive Review of Detection, Diagnosis, and Treatment Planning

**DOI:** 10.7759/cureus.70110

**Published:** 2024-09-24

**Authors:** Paschyanti R Kasat, Shivali V Kashikar, Pratapsingh Parihar, Pratiksha Sachani, Priyal Shrivastava, Smruti A Mapari, Utkarsh Pradeep, Gautam N Bedi, Paritosh N Bhangale

**Affiliations:** 1 Radiodiagnosis, Jawaharlal Nehru Medical College, Datta Meghe Institute of Higher Education & Research, Wardha, IND; 2 Obstetrics and Gynecology, Jawaharlal Nehru Medical College, Datta Meghe Institute of Higher Education & Research, Wardha, IND; 3 Medicine, Jawaharlal Nehru Medical College, Datta Meghe Institute of Higher Education & Research, Wardha, IND

**Keywords:** advanced imaging, early detection, metastatic epidural spinal cord compression, mri, pet-ct, treatment planning

## Abstract

Metastatic epidural spinal cord compression (MESCC) is a critical oncologic emergency caused by the invasion of metastatic tumors into the spinal epidural space, leading to compression of the spinal cord. If not promptly diagnosed and treated, MESCC can result in irreversible neurological deficits, including paralysis, significantly impacting the patient's quality of life. Early detection and timely intervention are crucial to prevent permanent damage. Imaging modalities play a pivotal role in the diagnosis, assessment of disease extent, and treatment planning for MESCC. Magnetic resonance imaging (MRI) is the current gold standard due to its superior ability to visualize the spinal cord, epidural space, and metastatic lesions. However, recent advances in imaging technologies have enhanced the detection and management of MESCC. Innovations such as functional MRI, diffusion-weighted imaging (DWI), and hybrid techniques like positron emission tomography-computed tomography (PET-CT) and PET-MRI have improved the accuracy of diagnosis, particularly in detecting early metastatic changes and guiding therapeutic interventions. This review provides a comprehensive analysis of the evolution of imaging techniques for MESCC, focusing on their roles in detection, diagnosis, and treatment planning. It also discusses the impact of these advances on clinical outcomes and future research directions in imaging modalities for MESCC. Understanding these advancements is critical for optimizing the management of MESCC and improving patient prognosis.

## Introduction and background

Metastatic epidural spinal cord compression (MESCC) is a serious complication of cancer that occurs when metastatic tumors invade the spinal vertebrae, compressing the epidural space and, ultimately, the spinal cord [1]. This compression can lead to irreversible neurological damage, loss of motor and sensory function, and severe pain. MESCC is considered an oncologic emergency, as delays in diagnosis and treatment can result in permanent disability, paralysis, and a significant reduction in the quality of life [2]. The condition typically arises in the advanced stages of cancer, particularly in patients with malignancies that have a propensity for bone metastasis, such as breast, prostate, lung, and renal cancers [3]. MESCC is a relatively common condition in patients with metastatic disease, affecting approximately 5% to 10% of the cancer patients [4]. Most cases are found in the thoracic spine, followed by the lumbar and cervical regions. Early recognition is crucial, as the natural progression of MESCC can lead to devastating outcomes, including permanent paralysis. Despite advances in cancer treatment, the incidence of MESCC has not significantly decreased, given the extended survival of patients with metastatic cancer. As a result, the clinical burden remains high, and timely intervention is essential to prevent further neurological deterioration [5].

The early detection of MESCC is vital for preserving neurological function and improving patient outcomes. Prompt diagnosis allows for timely therapeutic interventions, which may include surgery, radiation therapy, or a combination of both, aimed at decompressing the spinal cord and stabilizing the spine [6]. The window of opportunity for preserving neurological function is often narrow; once significant damage occurs, recovery may be incomplete, even with optimal treatment. For this reason, early diagnosis using advanced imaging techniques has become a cornerstone in the management of MESCC [7]. Imaging is indispensable in the detection, diagnosis, and treatment planning of MESCC. Traditionally, modalities like X-rays and computed tomography (CT) scans were employed to assess bony involvement, but they lacked the sensitivity required to visualize soft tissue and epidural space invasion [8]. Magnetic resonance imaging (MRI), which has emerged as the gold standard, provides detailed visualization of the spinal cord, tumor, and surrounding soft tissues, allowing for precise diagnosis and comprehensive evaluation of the extent of compression [9].

In recent years, advances in imaging technology have revolutionized the detection and management of MESCC. Functional MRI, diffusion-weighted imaging (DWI), and diffusion tensor imaging (DTI) offer enhanced sensitivity for detecting early cord changes [10]. Additionally, hybrid modalities like positron emission tomography-computed tomography (PET-CT) and PET-MRI combine functional and anatomical data, providing a more comprehensive understanding of tumor spread and metabolic activity. These advances not only improve diagnostic accuracy but also play a pivotal role in treatment planning, guiding surgical resection, and radiation therapy [11]. This comprehensive review explores the recent advances in imaging techniques for the detection, diagnosis, and treatment planning of MESCC. By examining the strengths and limitations of traditional imaging methods and highlighting innovations, this review seeks to provide an updated overview of how imaging has evolved in managing this critical condition. Additionally, it will discuss the impact of these advances on patient outcomes, the role of imaging in treatment planning, and future directions in the field. Ultimately, the goal is to offer clinicians and researchers a detailed understanding of the current state and potential future imaging applications in MESCC.

## Review

Pathophysiology of MESCC

Metastatic epidural spinal cord compression (MESCC) is a critical oncological emergency characterized by the compression of the spinal cord due to metastatic lesions in the epidural space [1]. Metastasis to the spine primarily occurs through hematogenous spread, lymphatic channels, or direct extension from adjacent structures. The vertebral venous plexus plays a pivotal role in this process, offering a direct pathway for tumor cells to invade the spinal canal due to its high vascularity and lack of valves, facilitating the retrograde flow of tumor cells from distant sites [12]. Moreover, certain tumors possess biological traits that enhance their metastatic potential, such as high angiogenic capacity, promoting new blood vessel formation. Tumor cells can also evade the immune system, enabling them to survive and proliferate once they reach the spinal region [13]. Anatomically, understanding MESCC requires knowledge of the spinal canal and epidural space. The spinal canal, which houses the spinal cord, is encased by bony structures, making it vulnerable to compression from extrinsic sources like tumors [14]. The epidural space between the dura mater and the vertebral column contains fat and blood vessels and is a common site for metastatic lesions. Tumors in this space can exert pressure on the spinal cord, leading to neurological deficits. The pathophysiology of MESCC progresses through several stages, beginning with vasogenic edema due to tumor-related venous obstruction, followed by mechanical compression, and ultimately leading to ischemia and potential infarction of spinal cord tissue [14]. Several primary tumors are frequently associated with MESCC, with breast cancer being one of the leading causes, particularly affecting the thoracic spine. Lung cancer is another major contributor, especially in the thoracic and lumbar regions, while prostate cancer commonly metastasizes to the spine, resulting in significant morbidity [15]. Multiple myeloma often involves the vertebrae, causing compression due to plasmacytoma formation. Other tumors, such as renal cell carcinoma and gastrointestinal cancers, can also lead to MESCC, though less commonly. Understanding the pathophysiology, including the mechanisms of metastatic spread, anatomical considerations, and the prevalence of certain malignancies, is essential for timely diagnosis and effective management of this critical condition [16].

Clinical presentation and diagnostic challenges

MESCC typically manifests with a range of symptoms, with back pain being the most prevalent, affecting 80%-95% of patients at the time of diagnosis. This pain is often severe, may worsen at night, and can be exacerbated by certain movements such as coughing, sneezing, or straining [1]. In addition to back pain, patients may present with neurological symptoms, including numbness, heaviness, or weakness in the arms or legs. Some individuals report a band-like sensation of pain around the chest or abdomen, changes in sensory perception such as tingling or electric shock sensations, and numbness in the saddle area. Furthermore, bowel or bladder control difficulties may arise, signaling significant spinal cord involvement. MESCC most commonly affects the thoracic spine (60%-70% of the cases), followed by the lumbosacral spine (20%-30%) and cervical spine (10%), with the location of pain sometimes providing diagnostic clues [17]. While MESCC is a critical consideration in cancer patients presenting with back pain and neurological deficits, it is essential to differentiate it from other potential causes. Osteoporotic compression fractures, particularly in older patients, are a common alternative diagnosis, as they can also cause significant thoracic back pain [2]. Intramedullary metastases, although less common than epidural metastases, may present with similar symptoms, including pain, weakness, and sensory changes. Additionally, leptomeningeal metastases can manifest as cauda equina syndrome, characterized by back pain and lower extremity weakness. These differential diagnoses underscore the importance of a thorough clinical evaluation and consideration of the patient's cancer history [18]. Relying solely on physical examination to diagnose MESCC has significant limitations. While clinical signs and symptoms can raise suspicion, neurological deficits may not appear until substantial spinal cord compression has occurred. This delayed presentation can lead to misdiagnosis or delayed treatment, potentially resulting in irreversible neurological damage. Moreover, the location of pain does not always correlate with the level of spinal cord compression, complicating the diagnostic process [19]. Therefore, maintaining a high index of suspicion based on clinical presentation, combined with prompt imaging - particularly MRI - is crucial for the early diagnosis of MESCC. This approach ensures timely intervention, vital for preserving neurological function and improving patient outcomes [20]. Clinical presentation and diagnostic challenges in MESCC are summarized in Table 1.

**Table 1:** Clinical presentation and diagnostic challenges in metastatic epidural spinal cord compression

Clinical Presentation	Description	Diagnostic Challenges
Localized back pain [5]	The most common initial symptom is usually progressive and localized to the site of spinal metastasis.	Often mistaken for musculoskeletal pain, delaying the diagnosis.
Radiculopathy [21]	Nerve root compression may cause radiating pain or sensory deficits along the nerve distribution.	Symptoms overlap with other spinal pathologies, complicating early detection.
Motor weakness [22]	Progressive motor deficits in the lower extremities, potentially leading to paralysis.	Neurological signs may develop insidiously, and weakness might be attributed to other causes like stroke.
Sensory loss [23]	Loss of sensation, particularly in a "stocking-glove" distribution.	Sensory changes may be subtle and under-reported by patients, especially in the elderly.
Bladder and bowel dysfunction [24]	Loss of control over bladder or bowel function due to spinal cord compression.	Often, it is a late sign of significant compression, making early intervention difficult.
Gait instability [25]	Difficulty in walking, often leading to falls or inability to ambulate.	It can be attributed to generalized weakness or peripheral neuropathy, delaying specific imaging for diagnosis.
Paraparesis/paraplegia [26]	Bilateral weakness of the lower extremities or complete loss of motor function.	Paraparesis can develop slowly, and early stages may be overlooked in routine clinical assessments.
Non-specific systemic symptoms [4]	Fatigue, weight loss, and other signs of metastatic disease.	Systemic symptoms can mask the underlying spinal compression and delay imaging focused on the spine.

Traditional imaging modalities in MESCC

**Figure 1:**
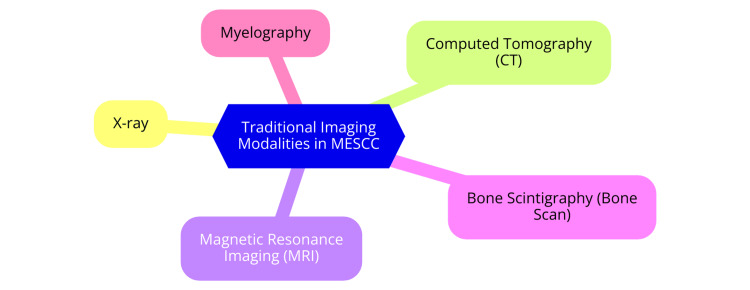
Traditional imaging modalities for MESCC Image credit: Dr. Paschyanti R. Kasat

MESCC is a serious condition that requires precise imaging for effective diagnosis and treatment. This section reviews conventional imaging techniques, including X-ray, computed tomography (CT), and magnetic resonance imaging (MRI), outlining their roles, advantages, and limitations [14]. X-rays have traditionally been the first choice for skeletal evaluations due to their accessibility and rapid results. However, their utility in diagnosing MESCC is limited. X-rays mainly reveal bony structures and may overlook subtle soft tissue metastasis-related changes [27]. They lack sensitivity for detecting early epidural disease or assessing the extent of spinal cord compression, making them inadequate for a thorough evaluation of MESCC. Although X-rays can identify significant bony metastases, their sensitivity is relatively low, especially for early-stage lesions. Small or subtle bony metastases often go undetected, so while X-rays can offer initial insights, they are unreliable for a definitive diagnosis in MESCC [2]. CT scans significantly surpass the X-rays in visualizing bony structures and assessing cortical integrity. They provide detailed images of the vertebrae, allowing for the detection of aggressive lesions and bony remodeling. CT is particularly valuable for evaluating bone involvement in patients with known malignancies and can identify pathological fractures related to metastatic disease [28]. Often used as an initial screening tool for patients with known cancer who present with neurological symptoms, CT quickly assesses the spine for bony metastases and is especially useful in emergencies due to its rapid acquisition. Contrast-enhanced CT can further improve the visualization of soft tissue and vascular anatomy, assisting in evaluating epidural masses and their impact on the spinal cord. While CT can help distinguish between benign and malignant lesions, it is less sensitive than MRI for soft tissue evaluation [29]. MRI is considered the gold standard for diagnosing MESCC because of its superior soft tissue contrast resolution. It accurately assesses the spinal cord, epidural space, and surrounding soft tissues, making it the preferred imaging modality for suspected MESCC. Various MRI sequences, such as T1-weighted, T2-weighted, and short tau inversion recovery (STIR), are used to visualize different aspects of MESCC. T2-weighted images are particularly effective for assessing edema and cord compression, while T1-weighted images offer detailed anatomical views of the spinal cord and surrounding structures [30]. STIR sequences are particularly useful for suppressing fat signals and enhancing the visibility of edema related to tumor infiltration. MRI shows high sensitivity in detecting soft tissue involvement, epidural masses, and the degree of spinal cord compression. Research indicates that MRI has significantly higher diagnostic accuracy than CT for detecting spinal osseous metastases, making it essential to manage MESCC [31]. Traditional imaging modalities for MESCC are illustrated in Figure 1.

Advances in imaging modalities for MESCC

Functional MRI (fMRI) has become an important tool for visualizing neurological changes and assessing the impact of spinal cord compression in patients with metastatic epidural spinal cord compression (MESCC) [32]. By detecting variations in blood oxygenation levels, fMRI can pinpoint spinal cord regions affected by compression and track changes over time. This information is vital for treatment planning, helping clinicians determine the extent of necessary surgical decompression or optimize radiation therapy targeting. The capability to visualize functional changes in the spinal cord improves the understanding of how compression influences neurological function, leading to more informed clinical decisions [33]. Diffusion-weighted imaging (DWI) and diffusion tensor imaging (DTI) are advanced MRI techniques that significantly enhance the detection of early spinal cord changes and metastatic lesions. These methods offer insights into the diffusion of water molecules within the spinal cord, which can be disrupted by compression or tumor cell infiltration. DWI and DTI have shown promise in predicting prognosis and treatment response, as alterations in diffusion parameters may precede visible structural changes seen on conventional MRI. These imaging techniques can enable timely interventions by detecting early microstructural changes, potentially improving patient outcomes [34]. Positron emission tomography-computed tomography (PET-CT) merges metabolic and anatomical imaging, allowing metastatic disease detection beyond the spine. The PET component identifies areas of increased metabolic activity that may indicate tumor cells, while the CT component provides detailed anatomical information. This comprehensive assessment assists in staging and treatment planning for patients with MESCC, offering a holistic view of the disease. By identifying metastases in other parts of the body, PET-CT scans inform decisions regarding systemic therapies and overall management strategies [35]. PET-MRI combines the functional and morphological imaging capabilities of PET and MRI, respectively. By integrating the superior soft tissue contrast of MRI with the high sensitivity of PET for early lesion detection, PET-MRI offers enhanced diagnostic accuracy and improved monitoring of treatment response in patients with MESCC. This hybrid imaging technique provides a more comprehensive evaluation of the structural and functional aspects of both the spinal cord and surrounding tissues, facilitating better treatment planning and follow-up assessments [36]. Emerging CT techniques, such as dual-energy and spectral imaging, are being explored for their potential in detecting MESCC. These advanced CT modalities can provide information on tumor composition and bone marrow involvement, which may assist in treatment planning and response assessment. These techniques improve the overall diagnostic process by characterizing the lesion nature and evaluating bone involvement, enabling more tailored treatment approaches [37]. Advances in imaging modalities for metastatic epidural spinal cord compression (MESCC) are summarized in Table 2.

**Table 2:** Advances in imaging modalities for metastatic epidural spinal cord compression (MESCC)

Imaging Modality	Key Features	Advantages	Limitations
Magnetic resonance imaging (MRI) [38]	The gold standard for detecting MESCC provides high-resolution images of soft tissues.	Superior soft tissue contrast, excellent visualization of spinal cord, nerve roots, and metastasis.	Limited availability, long scan times, contraindicated in patients with metal implants.
Computed tomography (CT) [39]	It provides detailed bone visualization and is often used with contrast.	Rapid imaging is excellent for detecting bone involvement and is useful for treatment planning.	Limited soft tissue resolution and high radiation dose compared to MRI.
Positron emission tomography-CT (PET-CT) [40]	Combines metabolic activity imaging with anatomical detail from CT.	It detects metabolically active lesions and is useful for identifying occult metastases.	High radiation exposure is less effective in detecting small lesions or early cord compression.
Diffusion-weighted imaging (DWI) [41]	MRI technique that highlights differences in tissue water mobility.	Sensitive to early spinal cord changes before anatomical changes are visible.	Susceptible to artifacts; it may not provide clear anatomical detail.
Single-photon emission computed tomography (SPECT) [42]	Functional imaging that detects abnormal bone metabolism.	Enhanced detection of metastatic bone lesions, especially in areas missed by conventional imaging.	Lower resolution compared to CT and MRI, limited soft tissue detail.
Dynamic contrast-enhanced MRI (DCE-MRI) [43]	Measures vascular permeability and blood flow in spinal metastases.	It helps in assessing the aggressiveness of the tumor and is useful for treatment planning.	It requires a contrast agent, is time-consuming and is less widely available than conventional MRI.
Ultrasound [44]	Portable imaging for superficial tissues and guidance for procedures.	Real-time imaging is non-invasive, and there is no radiation exposure.	Limited in deep tissue and spinal cord imaging, operator-dependent.
Functional MRI (fMRI) [45]	Assesses neural activity by detecting blood oxygenation changes.	Allows visualization of spinal cord function and neural pathways.	Mainly used for research, it is not widely available in clinical practice for MESCC diagnosis.
Hybrid PET-MRI [46]	Combines metabolic activity from PET with soft tissue detail from MRI.	It is excellent for detecting bone and soft tissue involvement and provides metabolic and anatomical data.	Expensive, limited availability, longer scan times.

Role of imaging in treatment planning

Imaging is pivotal in the treatment planning for MESCC, as it informs decisions related to surgical interventions, radiation therapy, and minimally invasive procedures. Advanced imaging techniques are crucial for optimizing patient outcomes and ensuring effective treatment [14]. Preoperative imaging is essential for planning surgical interventions in MESCC. Magnetic resonance imaging (MRI) is the gold standard for visualizing the extent of spinal cord compression and understanding the relationship of metastatic lesions to surrounding structures. MRI provides detailed information about the size, location, and characteristics of tumors, which helps surgeons determine the most effective approach for resection or decompression. In addition to MRI, CT scans may be used to evaluate bony involvement and guide stabilization strategies, offering a comprehensive view of the surgical landscape [47]. Advanced imaging techniques, such as three-dimensional (3D) reconstructions, significantly enhance surgical planning by providing precise anatomical visualization. These 3D models help surgeons understand the spatial relationships between tumors and critical structures. Intraoperative imaging guidance, such as fluoroscopy or CT, supports real-time navigation during surgery, improving tumor localization accuracy and procedural safety. This multimodal approach facilitates high-precision interventions, reducing complication risks and optimizing surgical outcomes [48]. Imaging is also crucial for delineating target volumes for radiation therapy in MESCC. MRI and CT define the tumor's extent and determine the treatment areas, ensuring that radiation is accurately delivered to the affected regions while sparing healthy tissue. This precision is essential for maximizing therapeutic effects and minimizing side effects [49].

Moreover, MRI and PET-CT are important for stereotactic body radiation therapy (SBRT). MRI provides high-resolution images for precise targeting, while PET-CT assesses metabolic activity, helping to distinguish between viable tumor tissue and necrotic areas. This information is vital for optimizing dose planning and treatment efficacy. Post-treatment follow-up imaging is crucial for evaluating the response to therapy, monitoring for recurrence, and adjusting future treatment plans as needed [50]. Imaging guidance is also integral to minimally invasive procedures such as vertebroplasty and kyphoplasty, which stabilize vertebral fractures caused by metastatic lesions. Techniques like fluoroscopy or CT are used to accurately position needles and inject cement into the vertebrae, enhancing procedural safety and effectiveness [51]. These minimally invasive methods often result in improved patient comfort and quicker recovery times compared to traditional surgical approaches. Emerging intraoperative imaging technologies, including advanced ultrasound and hybrid imaging systems, improve the precision of surgical interventions. These technologies enable real-time assessment of the surgical field, enhancing the ability to navigate complex anatomical structures and ensuring complete tumor resection while preserving neurological function. As these imaging modalities continue to evolve, they hold the potential to further refine treatment strategies for patients with MESCC [52]. The role of imaging in treatment planning for MESCC is summarized in Table 3.

**Table 3:** The role of imaging in treatment planning for MESCC MESCC: Metastatic epidural spinal cord compression

Imaging Modality	Role in Treatment Planning	Clinical Application	Limitations
MRI [39]	Provides detailed visualization of the spinal cord, nerve roots, and soft tissues.	Crucial for determining the extent of spinal cord compression, tumor involvement, and surgical planning.	Limited availability, contraindicated for patients with metal implants, and long scan times.
CT [31]	Excellent for assessing bone involvement and vertebral stability.	Used to assess the spine's structural integrity, plan surgical approaches, and guide radiation therapy.	Lower soft tissue resolution and high radiation exposure compared to MRI.
PET-CT [53]	Identifies metabolically active tumors and systemic metastasis.	Helps determine tumor burden, detect distant metastases, and guide systemic therapy decisions.	High radiation exposure, limited ability to differentiate between scar tissue and active disease.
SPECT (single-photon emission computed tomography) [54]	Functional imaging for detecting abnormal bone metabolism.	Useful for detecting skeletal metastases, especially in cases where MRI or CT are inconclusive.	Lower resolution is not commonly used for soft tissue or detailed structural assessment.
DCE-MRI (dynamic contrast-enhanced MRI) [55]	Provides information on tumor vascularity and perfusion.	Aids in assessing tumor aggressiveness and predicting response to therapies such as radiation or surgery.	Requires contrast administration, is more time-consuming, and is not widely available compared to conventional MRI.
DWI (diffusion-weighted imaging) [56]	Detects early spinal cord changes, assessing tumor infiltration.	Useful in differentiating between malignant and benign lesions, aiding in preoperative planning.	Prone to artifacts and less effective in differentiating between tumor types in the spine.
Ultrasound [57]	Used for procedural guidance in real time, such as biopsies or pain management injections.	It supports image-guided interventions and the assessment of superficial lesions in selected cases.	Limited in deep tissue or spine visualization, mostly used for adjunctive procedures.
fMRI (functional MRI) [58]	Assesses spinal cord function and neural pathways, providing functional mapping for surgeries.	It helps in preoperative planning to avoid damage to functional areas and is useful in high-risk surgical cases.	Primarily research-based and not yet widely available for routine clinical treatment planning in MESCC cases.
CT myelography [9]	Combines CT with contrast dye in the spinal canal for detailed spinal cord imaging.	Useful in patients who cannot undergo MRI; it provides a detailed visualization of nerve root compression.	Invasive, using contrast dye and radiation, limited soft tissue resolution compared to MRI.

Future directions in imaging for MESCC

Integrating artificial intelligence (AI) and machine learning into imaging for MESCC offers significant potential for enhancing detection and diagnosis. Advanced deep learning models have shown promise in automating the identification and classification of MESCC on MRI scans, providing radiologists with powerful tools to accelerate diagnosis [59]. These models can accurately evaluate the severity of spinal cord compression, which is critical for timely intervention. As AI technology evolves, we expect the development of more sophisticated algorithms capable of analyzing a broader range of imaging findings. This progress could lead to improved decision support systems that help clinicians determine the most appropriate treatment pathways for patients [59]. In addition to enhancing diagnostic capabilities, predictive imaging models are emerging as crucial tools for assessing treatment response and long-term outcomes in MESCC patients. Advanced imaging techniques, such as diffusion tensor imaging (DTI) and dynamic contrast-enhanced (DCE) MRI, can quantify key parameters like spinal cord perfusion and diffusion [60]. These imaging biomarkers have the potential to predict patient responses to specific treatments, enabling more personalized and effective therapeutic strategies. By identifying patients at higher risk for poor functional outcomes, healthcare providers can tailor interventions to optimize care and improve overall survival rates [60].

The future of MESCC imaging also involves integrating imaging data with biomarker and genomic information. Combining traditional imaging modalities with emerging biomarkers, such as circulating tumor cells or cell-free DNA, and genomic profiling of tumors can offer a more comprehensive understanding of disease biology [61]. This multimodal approach allows clinicians to stratify patients based on their risk of developing MESCC and guide personalized treatment strategies. By correlating imaging findings with molecular characteristics, we can enhance our ability to predict disease progression and treatment efficacy, improving patient outcomes [61]. Emerging technologies, such as interventional MRI and intraoperative imaging, provide opportunities for real-time monitoring of MESCC progression and treatment response. These innovative techniques could enable clinicians to assess the effectiveness of surgical interventions as they occur, ensuring adequate decompression and prompt detection of any signs of recurrence [62]. Real-time imaging capabilities can facilitate timely adjustments to treatment plans, improving the overall management of MESCC. Integrating dynamic monitoring into clinical practice can enhance decision-making processes and better address the evolving needs of patients with this complex condition [62]. Future directions in imaging for MESCC are summarized in Table 4.

**Table 4:** Future directions in imaging for metastatic epidural spinal cord compression (MESCC)

Imaging Technology	Future Potential	Key Advancements	Challenges
Artificial Intelligence (AI) and Machine Learning [63]	AI algorithms for automated detection and segmentation of spinal cord compression.	Improved accuracy and faster diagnosis, aiding in early intervention and personalized treatment plans.	Requires extensive training data, regulatory challenges, and clinical validation for widespread adoption.
Radiomics [64]	Extracting quantitative data from medical images for personalized diagnosis.	Allows for prediction of tumor behavior, prognosis, and treatment response based on imaging characteristics.	High complexity requires advanced software and integration with clinical workflows and interpretability.
High-resolution MRI [65]	Enhancements in MRI resolution for better tissue differentiation and early detection.	Increased precision in visualizing spinal cord compression, nerve root involvement, and tumor boundaries.	High cost, limited availability in clinical settings, and longer scan times.
4D MRI [66]	Captures dynamic imaging over time to assess tumor and spinal cord motion.	Helps in monitoring tumor growth, progression, and response to treatments like radiation or surgery.	Limited access, complex image interpretation, and longer imaging times compared to conventional MRI.
Hybrid PET-MRI [67]	Combining functional and anatomical imaging for detailed tumor and cord visualization.	Potential to simultaneously assess metabolic activity and anatomical structures, improving treatment planning.	Expensive, limited access and longer imaging times require specialized image interpretation expertise.
Molecular Imaging [68]	Detects molecular changes at the cellular level before structural changes appear.	Could enable ultra-early diagnosis and targeted treatment of spinal metastasis.	Still in experimental stages, requires extensive research and development, and may involve high costs.
Advanced ultrasound techniques [69]	High-resolution ultrasound with elastography for spinal imaging.	It could provide real-time imaging for guided interventions and improved detection of superficial metastases.	Limited depth penetration is operator-dependent and primarily useful as an adjunct rather than a standalone.
Functional MRI (fMRI) with neuro-navigation [70]	Combining fMRI with real-time neuro-navigation for precise surgical planning.	Allows for intraoperative monitoring of neural pathways, reducing surgical risks in delicate areas.	High cost, requires specialized equipment and training, and is limited to high-end centers.
Hyperpolarized MRI [71]	Enhances signal-to-noise ratio for improved tissue contrast and metabolic imaging.	Provides detailed information on tumor metabolism and spinal cord condition, potentially guiding therapy.	Still experimental, high cost, and limited to research settings.
Optical coherence tomography (OCT) [72]	Non-invasive imaging at a microscopic level for the real-time assessment of tissues.	It may allow for intraoperative, real-time guidance in delicate spinal surgeries or biopsies.	Early-stage development is limited to research and requires integration with existing surgical tools.

## Conclusions

In conclusion, advancements in imaging have significantly transformed the detection, diagnosis, and treatment planning of metastatic epidural spinal cord compression (MESCC), offering critical improvements in patient outcomes. Though still useful for assessing bone involvement, traditional modalities such as X-ray and CT scans have been surpassed by MRI as the gold standard for visualizing soft tissues and the extent of spinal cord compression. The emergence of newer techniques, such as functional MRI, diffusion-weighted imaging, and hybrid modalities like PET-CT and PET-MRI, has further enhanced the sensitivity and specificity of imaging, allowing for earlier detection and more precise treatment planning. These innovations have improved diagnostic accuracy and contributed to better surgical and radiation therapy outcomes by providing detailed anatomical and functional insights. As imaging continues to evolve, future developments in artificial intelligence and real-time imaging hold promise for even more personalized and efficient care. This review underscores the essential role of cutting-edge imaging in the timely management of MESCC, highlighting its impact on clinical practice and the patient's quality of life.
